# Electroconvulsive Therapy for Obsessive-Compulsive Symptoms in Schizophrenia Spectrum Disorders: A Systematic Review

**DOI:** 10.7759/cureus.93675

**Published:** 2025-10-01

**Authors:** Mohamad W Midou, Abhiraj Sanku, Austin Lui, James Eyerman

**Affiliations:** 1 College of Osteopathic Medicine, Touro University California, Vallejo, USA; 2 Psychiatry, College of Osteopathic Medicine, Touro University California, Vallejo, USA

**Keywords:** ect, electroconvulsive therapy, obsessive compulsive, obsessive-compulsive disorder, ocd, psychiatry, psychotic, schizo-obsessive disorder, schizophrenia, schizophrenic

## Abstract

Obsessive-compulsive symptoms (OCS) and obsessive-compulsive disorder (OCD) can co-occur with schizophrenia spectrum disorders. There is limited research on electroconvulsive therapy (ECT) for this particular comorbidity. This systematic review assessed the efficacy of ECT for OCS/OCD in patients with schizophrenia spectrum disorders. A systematic literature search was conducted using multiple databases. Inclusion criteria for articles included those involving patients with both OCS/OCD and schizophrenia spectrum disorders treated with ECT. Out of 10 studies, 11 patients met the inclusion criteria. Nine were case reports and one was a retrospective study. Seven out of 11 patients experienced improvement in OCS after ECT, including four with complete remission. Additionally, two acutely suicidal patients due to OCS/OCD had remission of suicidality. The mean reduction in Yale-Brown Obsessive Compulsive Scale scores across four patients was 29.5±7.7. Maintenance ECT was effective in preventing relapse in three patients. No worsening of symptoms or major adverse effects were reported. ECT may offer a potentially beneficial treatment option for OCS/OCD in patients with schizophrenia spectrum disorders, particularly those with treatment-resistant or severe symptoms. The small number of patients, predominance of case studies, and heterogeneous evidence limit conclusions and treatment guidance. Further studies are necessary to determine efficacy, adverse effects, and optimal use of ECT in this population.

## Introduction and background

Schizophrenia spectrum disorders are characterized by one or more of the following: delusions, hallucinations, disorganized thinking, disorganized or abnormal behavior, and negative symptoms [1]. These disorders include schizophrenia, other psychotic disorders, and schizotypal personality disorder [1]. Obsessive-compulsive disorder (OCD) is characterized by obsessions and/or compulsions [1]. Obsessions are recurrent unwanted thoughts, images, or urges that cause distress or significant anxiety, and compulsions are repetitive behaviors or mental acts in response to obsessions to relieve distress or anxiety [1]. Patients with schizophrenia spectrum disorders can have obsessive-compulsive symptoms (OCS) [2]. In some cases, OCS may be severe enough to meet criteria for comorbid obsessive-compulsive disorder (OCD) [2]. OCS/OCD in schizophrenia can develop during the prodromal psychotic state, in the first psychotic episode, with chronic schizophrenia, or after treatment with antipsychotics [2]. Case studies describe presentations with varying degrees of insight as described by the DSM-5-TR, ranging from obsessions clearly distinct from delusions with good/fair insight to obsessions with absent insight and delusional perseverations that feed into OCS/OCD [1,3]. 

Studies estimating the prevalence of OCS/OCD in schizophrenia have a wide range depending on the population studied, with comorbid OCS estimated at 13-51% and comorbid OCD estimated at 10-24% [4]. These prevalence rates are higher than in the general population [5]. The high prevalence is complicated by the association between second-generation antipsychotics and OCS, with clozapine, olanzapine, and risperidone being the most implicated [2,5,6]. This association may be caused by the anti-serotonergic effects of these medications [5]. However, OCS in schizophrenia is not entirely explained by antipsychotics, considering the high prevalence of OCS in the prodromal phase, in initial psychotic episodes, and before antipsychotic therapy [2].

Neurophysiological studies show significant differences between patients with schizophrenia with OCS and those without [2]. Neurophysiological findings of OCS in psychotic patients include the implication of serotonergic, dopaminergic, and glutaminergic neurotransmitter systems; multiple genetic factors; and abnormalities in brain regions including the frontal lobe, basal ganglia, thalamus, and cerebellum [7]. Based on the high prevalence, unique clinical picture, poor prognosis [8], and neurophysiological underpinnings, some authors postulate the “schizo-obsessive” presentation is a distinct subtype of schizophrenia, suggesting a unifying pathophysiology for the development of OCS/OCD in schizophrenia [3,9-11]. However, there remains no Diagnostic and Statistical Manual or International Classification of Diseases diagnosis specific to schizophrenia with OCS.

Patients with both schizophrenia and OCS have more severe psychotic symptoms and poorer quality of life [8], as well as increased suicidality [12]. Despite this, effective treatment options remain unclear. The American Psychiatric Association has suggested adding antidepressants to antipsychotics [13]. Fluvoxamine adjunct to antipsychotics is a well-studied therapy for OCS in schizophrenia [4]. Other pharmacotherapies with some evidence include olanzapine, ziprasidone, aripiprazole, or amisulpride [2,4]. Limited data suggest cognitive behavioral therapy can be useful [2].

Electroconvulsive therapy (ECT) has most often been used for depression, severe mania, catatonia, and treatment-resistant schizophrenia [14]. The treatment involves the administration of a controlled electrical current to the patient's brain via scalp electrodes to induce a therapeutic seizure with anesthetic sedation and pharmacologic augmentation [14]. Acute ECT is a course of ECT given to rapidly alleviate the target symptoms. Maintenance ECT consists of additional ECT over a longer period of time and with lower frequency, which can be effective in preventing relapse of symptoms [15]. ECT has been shown to impact genetic expression and neurochemical signaling, contributing to changes in brain neural networks and physiology [16]. Contraindications to ECT include pheochromocytoma, elevated intracranial pressure, cardiovascular conduction defects, high-risk pregnancies, and aortic and cerebral aneurysms [17]. ECT research in treatment-resistant schizophrenia has shown significant benefit, especially in non-responders to medication management [18]. There is some evidence for a positive response from ECT in OCD; however, no randomized controlled studies have been conducted to adequately assess its efficacy for OCD [19].

Given the high prevalence and increased severity of OCS/OCD in schizophrenia spectrum disorders, along with the effectiveness of ECT in treatment-resistant schizophrenia, ECT for OCS/OCD in schizophrenia may represent a promising treatment approach worth investigating. This review seeks to assess the current literature for studies on this topic. As already mentioned, literature on this topic is limited and is composed mostly of case studies. A systematic review is still an appropriate study type for a poorly reported topic despite the increased risk of bias in case studies [20]. This review can provide a summary of the present research, guide future directions of study, and offer important information for clinicians. 

## Review

Methods

This systematic review followed the Preferred Reporting Items for Systematic Reviews and Meta-Analyses (PRISMA) guidelines [21]. The review protocol was not registered. A meta-analysis was not conducted due to clinical heterogeneity among the studies included and the limited number of patients. The study investigators independently performed title and abstract screening for relevant articles based on inclusion and exclusion criteria. The full texts of each selected article were obtained, reviewed, screened further based on inclusion and exclusion criteria, and checked for relevant references.

Search Strategy

Relevant MeSH and pertinent free-text keywords were used to capture studies involving schizophrenia spectrum disorders and OCS/OCD with ECT. No filter for date of publication was applied. Academic Search Complete, APA PsycInfo, Embase, Medline, and PubMed were systematically searched with the terms: ("schizophrenia" OR "schizophrenic" OR "schizophreniform" OR "psychotic" OR "psychosis" OR "delusional") AND ("obsessive-compulsive" OR "obsessive" OR "compulsive" OR “OCD”) AND ("electroconvulsive" OR "ECT"). Additionally, a grey literature search was performed using Google Scholar with the keywords above. Sources were last searched on 28 May 2025. 

Eligibility Criteria

The inclusion criteria were: (1) a published case report, case series, observational study, or clinical trial in English; (2) at least one patient with schizophrenia spectrum disorder, including schizophrenia, schizophreniform disorder, brief psychotic disorder, delusional disorder, or schizoaffective disorder; (3) the patient(s) had OCS or OCD; and (4) the patient(s) received ECT. Studies were excluded if they did not report the impact of ECT on OCS/OCD, were non-human studies, involved psychosis secondary to organic conditions, or did not present original patient data. 

Quality Assessments

The risk of bias in non-randomized observational studies was assessed by the investigators with the ROBINS-I tool [22] by two of the authors. For case studies, the Joanna Briggs Institute Critical Appraisal Checklist for Case Reports [23] was used for overall quality assessment by two of the authors. Disagreements in quality assessments were resolved through discussion with the other authors. Reporting bias and certainty were assessed narratively by the authors.

Data Collection

The primary outcome assessed was the change in severity of OCS/OCD following ECT. Outcomes for OCS/OCD, as well as for suicidal ideation and catatonic symptoms, were obtained from clinical description, clinician assessment, patient report, and use of standardized rating scales for OCS (e.g., Yale-Brown Obsessive Compulsive Scale [Y-BOCS] [24]). Definitions for treatment response and remission were based on guidelines proposed by Mataix-Cols et al., which include conceptual and operational definitions [25]. Other data collected were the nation of origin of the study, age, sex, gender, psychiatric diagnoses, symptoms, symptom onset, type and frequency of ECT, and concurrent medications. Sex and gender were noted as reported by the study authors. Means and percentages were calculated for relevant data points collected from the studies. Percentages may not sum to 100% due to rounding. The mean and mean difference with standard deviation of Y-BOCS scores were calculated using the scores reported in the included studies. This was a descriptive synthesis without statistical pooling due to limited data.

Results

Study Selection

**Figure 1:**
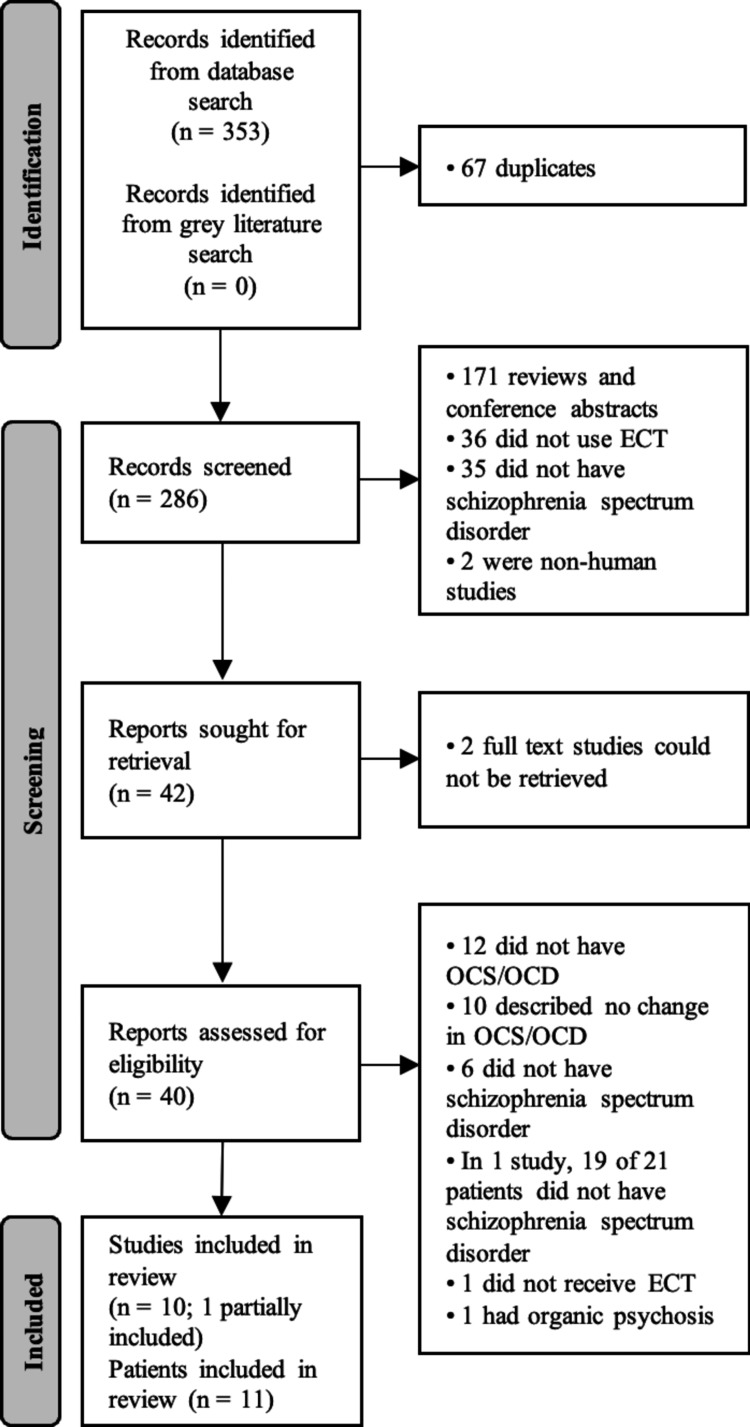
Flow diagram of the selection process OCS: obsessive-compulsive symptoms; OCD: obsessive-compulsive disorder; ECT: electroconvulsive therapy

The database search yielded 353 articles published in English. No relevant articles were found with the grey literature search. Sixty-seven of these were duplicates, leaving 286 studies for screening for inclusion via title and abstract. Of these, 244 studies were excluded. The full texts of the remaining 42 studies were reviewed, and a final 10 studies met the inclusion criteria. The selection process is shown in Figure 1, and the details of the results are shown in Table 1. Nine of the 10 studies were single-patient case reports, and one was a retrospective observational study that included two relevant patients. This resulted in 11 total patients.

**Table 1:** Characteristics of the reviewed studies ++: remission; +: partial response; 0: no response NR: not reported *Yale-Brown Obsessive Compulsive Scale; ** Patient was trialed on multiple medication regimens during ECT OCS: obsessive-compulsive symptoms; OCD: obsessive-compulsive disorder; CR: case report; Retro.: retrospective study; B/L: bilateral; U/L: unilateral; MECT: maintenance ECT

Authors, year	Study design	Age & sex	Psychotic diagnosis	OCD diagnosis	OCS/OCD onset & characteristics	ECT characteristics	Concurrent medications	OCS outcome	Change in Y-BOCS^ *^
Bai et al., 2018 [26]	CR	35; male	Schizoaffective, depressive type, w/ catatonia	No	Onset during chronic schizophrenia; repetitive rituals	B/L 3x/wk for 3 wks	Risperidone 2 mg	+	NR
Chaves et al., 2005 [27]	CR	17; male	Schizophrenia	Yes	Onset prior to psychosis; violent obsessions/compulsions	B/L 2x/wk for 3 wks	None	+	-34
Haack et al., 2014 [28]	CR	18; male	Schizophrenia, w/ catatonia	No	Onset of initial psychotic episode; symmetry obsessions	B/L 3x/wk for 6 wks	Trialed:^**^ lorazepam, fluoxetine, olanzapine, clomipramine, aripiprazole, clozapine	0	NR
Hanisch et al., 2009 [29]	CR	48; female	Schizoaffective	No	Onset during chronic schizophrenia; contamination obsessions, repetitive rituals, hoarding, avoidance	U/L 2x/wk for 5 wks; MECT 1 mo later: 1x/2wks for 42 wks	During MECT: sertindole 12 mg, mirtazapine 45 mg	++	-34
Joshi et al., 2024 [30]	CR	26; female	Unspecified schizophrenia disorder	Yes	Onset prior to psychosis; contamination obsessions/compulsions, acutely suicidal	4 sessions then 6 sessions 2 wks later; MECT 1x/wk for 1 mo	Fluoxetine 80 mg, olanzapine 10 mg	++	-32
Lavin et al., 1996 [31]	CR	37; female	Schizophrenia	Yes	Unspecified onset; repetitive rituals	B/L 12 total sessions	None	++	NR
Lelliot et al., 1987 [32]	CR	24; male	Schizophrenia	Yes	Onset with initial psychotic episode; symmetry obsessions, repetitive rituals	8 U/L sessions then 6 U/L sessions ~30 wks later	Clomipramine 250 mg	++	NR
Li et al., 2022 [33]	Retro.	NR	Unspecified schizophrenia disorder	Yes	Unspecified onset and symptoms	B/L 3x/wk for unspecified duration	Unspecified	0	NR
Nayak et al., 2019 [34]	CR	43; male	Schizophrenia	No	Unspecified onset; repetitive rituals	Unspecified acute	Quetiapine 25 mg	0	NR
Rao et al., 2011 [35]	CR	40; female	Schizophrenia	No	Clozapine induced; sexual obsessions, cognitive compulsions, acutely suicidal	B/L 3x/wk for 8wks; MECT 1x/wk for 3 mo	Chlorpromazine 400 mg, escitalopram 40 mg, Flupenthixol 40 mg/2 wks IM	+	-18

Quality Assessments

Table 2 shows the bias assessment of the retrospective study. Table 3 shows the quality assessments of the case studies.

**Table 2:** ROBINS-I assessment of the retrospective study

Authors, year	Confounding	Selection of participants	Classification of interventions	Deviations from intended intervention	Missing data	Measurement of outcomes	Selection of reported result	Overall
Li et al., 2022 [33]	Serious	Moderate	Low	Low	Low	Moderate	Low	Serious

**Table 3:** Joanna Briggs Institute Checklist for Case Reports Y: yes; N: no

Authors, year	Demographic description	History provided	Clinical condition described	Diagnostic methods described	Intervention procedures	Post-intervention condition described	Adverse events reported	Takeaway lessons
Bai et al., 2018 [26]	Y	Y	Y	Y	Y	Y	Y	Y
Chaves et al., 2005 [27]	Y	Y	Y	Y	Y	Y	N	Y
Haack et al., 2014 [28]	Y	Y	Y	Y	Y	Y	Y	Y
Hanisch et al., 2009 [29]	Y	Y	Y	Y	Y	Y	Y	Y
Joshi et al., 2024 [30]	Y	Y	Y	Y	Y	Y	N	Y
Lavin et al., 1996 [31]	Y	Y	Y	N	Y	Y	N	Y
Lelliot et al., 1987 [32]	Y	Y	Y	Y	Y	Y	N	Y
Nayak et al., 2019 [34]	Y	Y	Y	Y	Y	Y	Y	Y
Rao et al., 2011 [35]	Y	Y	Y	Y	Y	Y	N	Y

Demographics

Five patients were male or men (45%), four were female or women (36%), and two were not described by sex or gender (18%) (those of the retrospective study). Patient ages ranged from 17 to 48 years old, with a mean of 32 years old.

Diagnoses and Symptoms

Six patients were diagnosed with schizophrenia (55%), two were diagnosed with schizoaffective disorder (18%), and three were diagnosed with unspecified schizophrenia spectrum disorders (27%). 

Of these patients, six were diagnosed with OCD (55%), while the other five (45%) exhibited significant OCS not meeting OCD criteria. Four patients had an unspecified onset of OCS/OCD (36%), two had OCS onset during the course of chronic schizophrenia (18%), two had OCS/OCD onset before developing psychosis (18%), two had OCS during their initial psychotic episode (18%), and one had OCS onset due to clozapine (9%). The most common symptoms were repetitive rituals (36%), symmetry obsessions (18%), and contamination obsessions and/or compulsions (18%). Other features included avoidance behaviors, hoarding, sexual obsessions, and violent obsessions and compulsions. 

Management

The primary indications for ECT were OCS/OCD in five patients (45%), both psychosis and OCS/OCD in four patients (36%), and catatonia in two patients (18%). The decision to use ECT was guided by the clinician's judgement. Seven of the patients were treated with bilateral (bitemporal) ECT (64%), two with unilateral ECT (18%), and two were not specified (18%). All patients received acute ECT. Five received acute ECT three times per week (45%), four did not specify frequency (36%), and two received acute ECT twice per week (18%). Two patients received a second course of acute ECT for relapse of symptoms (18%). Based on the available data, the mean number of ECT sessions in the acute setting was 10.3. Additionally, three patients received additional maintenance ECT after relapse of symptoms (27%).

All the patients with available pharmacologic data (82%) had multiple unsuccessful medication trials for OCS/OCD prior to receiving ECT. Concurrent with ECT, seven patients were given psychotropic medications (64%), two patients were given no medication treatment (18%), and two patients had an unknown medication treatment status (18%). These medications included both typical and atypical antipsychotics, benzodiazepines, selective serotonin reuptake inhibitors, tricyclic antidepressants, and mirtazapine.

Outcomes

Following acute ECT, seven patients (64%) had improvement of OCS/OCD, with four (36%) reaching complete remission and three (27%) with partial remission. The remaining four patients (36%) experienced no change in OCS/OCD. Of patients with improvement in OCS/OCD, five (71%) also received concurrent psychotropic medications. Three of the patients who experienced initial improvement from ECT (27%) relapsed with OCS/OCD and thus received maintenance ECT. All three eventually achieved sustained improvement after maintenance ECT. Initial OCS/OCD severity was assessed through Y-BOCS in four patients. Their mean Y-BOCS before ECT was 37±10.4. After ECT, the mean Y-BOCS was 7.5±5.7. The mean difference was -29.5±7.7. Additionally, two patients were found to have suicidal ideation that resolved after ECT. The two patients with catatonia also had resolution of catatonic symptoms. No worsening symptoms or adverse effects due to ECT were reported in any of the patients. 

Discussion

This review aimed to assess the efficacy of ECT for the treatment of OCS and OCD in people with schizophrenia spectrum disorders. Despite limited literature on this patient population, the findings suggest ECT may offer potential benefits for patients with schizophrenia spectrum disorder with treatment-resistant or severe OCS/OCD.

Most patients in this review received bilateral ECT, though frequencies and treatment durations varied. Seven out of eleven patients (64%) experienced improvement in OCS following ECT, and four of them achieved complete remission. The mean reduction in Y-BOCS scores was -29.5±7.7. In all four patients for whom Y-BOCS was reported, the reductions exceeded 35%, indicating clinically meaningful improvement [25]. These results show ECT may help improve OCS in schizophrenia spectrum disorders. 

Both patients with acute suicidal ideation related to OCS/OCD experienced remission of suicidal ideation with ECT. All patients had previously failed to respond to pharmacologic treatments for OCS/OCD before ECT. This further supports ECT as a possible treatment modality for patients with schizophrenia spectrum disorder with treatment-resistant OCS/OCD and/or severe OCS/OCD resulting in acute suicidal ideation. This is consistent with a review of ECT for primary OCD, which found that ECT was effective for patients with more severe symptoms [19]. Another study using a Swedish registry of 285 patients with schizophrenia found that a subset of 16 patients with comorbid OCD and schizophrenia had greater improvements in schizophrenia symptoms than non-comorbid patients after ECT, suggesting that comorbidity of psychiatric conditions may predict ECT effectiveness [36]. These findings support the utility of ECT in treatment-resistant schizophrenia spectrum disorder, particularly when symptoms are severe and complicated by psychiatric comorbidities. ECT may be particularly beneficial for patients with a “schizo-obsessive” presentation, who typically exhibit more severe symptoms and have worse prognoses.

In patients with relapse after initial improvement with ECT, subsequent ECT or maintenance ECT resulted in sustained improvement of symptoms. This further supports ECT for OCS/OCD in schizophrenia spectrum disorder, as well as the use of additional ECT in initial responders. The use of maintenance or continuation ECT in these cases mirrors its role in mood and psychotic disorders, where periodic booster sessions are commonly employed to prevent relapse and reduce hospitalization risk [37].

No consistent pattern was observed in those who responded to ECT in terms of onset of OCS/OCD or its characteristics, suggesting ECT may be effective for a variety of presentations and onset patterns, including clozapine-induced OCS. Also, ECT appeared to be effective regardless of its initial indication, furthering its applicability. This broad applicability may reflect ECT’s ability to modulate neural networks implicated in both disorders, specifically by decreasing activity of the cortico-striato-thalamo-cortical (CSTC) loop, which has been shown to be hyperactive and imbalanced in OCD and dysfunctional in schizophrenia spectrum disorders [38,39].

While most patients continued pharmacotherapy during ECT, no consistent medication treatment regimen was associated with outcomes. Notably, in relapsed cases, symptom improvement followed additional ECT rather than medication adjustments. Furthermore, these findings, along with the absence of reported adverse effects, suggest ECT is potentially useful and safe regardless of concurrent psychopharmacotherapy. This is particularly relevant given that cognitive-behavioral therapy, effective for OCD, is often limited in schizophrenia spectrum disorder due to impaired insight, working memory deficits, and disorganized thinking [40]. In such cases, ECT may offer a viable alternative that does not rely on a patient’s cognitive participation.

Among the four patients without improvement, there is no consistent pattern in symptoms, ECT regimen, or concurrent therapy to elucidate non-responsiveness. Importantly, there were no reported exacerbations in OCS/OCD from ECT, nor any other serious adverse events. Side effects, including prolonged seizures, transient confusion and amnesia, headaches, and cardiovascular abnormalities, have been reported in the acute post-ECT period [41]. However, no such events were described with the patients in this review. 

Short-term memory impairment may occur immediately after ECT; however, it does not appear to impair global cognition, attention, executive function, or long-term memory, including in patients with schizophrenia [42,43]. This is especially relevant in those with comorbid OCS/OCD, considering that studies show OCS/OCD impairs working memory and executive function [44,45]; the studied comorbidity may particularly impair abstract thinking [46]. ECT may improve functional outcomes and quality of life in schizophrenia, particularly through its positive effects on negative symptoms and cognition [42,47]. This has important implications for patients whose OCS/OCD symptoms interfere with daily living or treatment adherence. For patients with severe symptoms, ECT can offer therapeutic benefits independent of a patient’s capacity for engagement or adherence [48]. This makes ECT uniquely suited for cases where pharmacologic or behavioral interventions are not feasible.

Overall, these findings suggest that ECT, although historically used for affective or catatonic presentations, may offer a treatment option for patients with comorbid schizophrenia spectrum disorder and obsessive-compulsive symptoms. In particular, those with severe, treatment-resistant “schizo-obsessive” presentations may benefit most from ECT when standard therapies fail.

Limitations 

This review is limited by the small number of available studies and the predominance of case reports, which limit generalizability. Heterogeneity in study design, diagnostic criteria, ECT parameters, and outcome measures further complicates definitive conclusions. Though no studies reported serious adverse effects from ECT, only half of the case studies explicitly reported on adverse effects or lack thereof. Follow-up on symptom remission was limited in some included studies. Only four patients were assessed with standardized scales, and some lacked detailed reporting on concurrent treatments and treatment timelines. Furthermore, the retrospective study did not describe patient demographics, symptoms, disease onset, or medication regimens; this study was also found to be at serious risk of bias. This review may also be subject to publication bias, as case reports and smaller studies with positive outcomes are more likely to be published. The findings of this review are preliminary due to these limitations. 

Future Directions

Future research with more rigorous methods, standardized outcome measures, and larger sample sizes is needed to determine the efficacy of ECT in reducing OCS in this population. Future studies should use standardized pre- and post-intervention measures of OCS/OCD, such as the Y-BOCS. Also, standardized use of measures for suicidal ideation, such as the Suicidal Ideation Attributes Scale or Columbia-Suicide Severity Rating Scale, can be used. Consistent long-term follow-up for assessment of relapse rates and functional recovery is also needed. The lack of randomized controlled trials remains a significant gap. 

Future studies can investigate whether baseline clinical features such as symptom severity, neurophysiological circuit dysfunction, or response to medication therapy may predict responsiveness to ECT in this group. Other neuromodulation techniques like deep brain stimulation and transcranial magnetic stimulation may also be promising to explore, but remain less accessible.

## Conclusions

In this systematic review, ECT was found to improve OCS/OCD in seven out of the 11 patients with schizophrenia spectrum disorders studied, including four who achieved remission of OCS/OCD. No exacerbations or serious adverse effects from ECT were reported. Additionally, the two acutely suicidal patients due to OCS had remission of suicidality. All patients with available data had multiple unsuccessful medication trials before ECT. Therefore, ECT may potentially offer a beneficial treatment option for OCS/OCD in patients with schizophrenia spectrum disorders, particularly in those with treatment-resistant or severe symptoms. The small number of patients, predominance of case studies, and heterogeneous evidence limit conclusions and treatment guidance. Further studies are necessary to determine ECT’s efficacy, direct causality, mechanism of action, and optimal use for OCS/OCD in schizophrenia spectrum disorders.
